# Statistics anxiety and performance: blessings in disguise

**DOI:** 10.3389/fpsyg.2015.01116

**Published:** 2015-08-04

**Authors:** Daniel Macher, Ilona Papousek, Kai Ruggeri, Manuela Paechter

**Affiliations:** ^1^Educational Psychology Unit, Department of Psychology, University of GrazGraz, Austria; ^2^Biological Psychology Unit, Department of Psychology, University of GrazGraz, Austria; ^3^Department of Psychology, University of CambridgeCambridge, UK

**Keywords:** statistics anxiety, performance, state anxiety, expectancy-value theory, effort

## Introduction

Statistics anxiety describes the apprehension that occurs when an individual is exposed to statistics content or problems and instructional situations, or evaluative contexts that deal with statistics. As statistics-anxious individuals always experience anxiety when doing statistics, statistics anxiety describes an enduring, habitual type of anxiety (Onwuegbuzie and Wilson, 2003; Macher et al., 2011).

A large proportion of students identify statistics courses as the most anxiety-inducing courses in their curriculum (Zeidner, 1991). Especially in subjects such as psychology, education, or sociology, statistics anxiety is widely spread among students (Onwuegbuzie and Wilson, 2003; Onwuegbuzie, 2004; Ruggeri et al., 2008). These subjects are often chosen by students with less interest and more critical self-assessments in mathematics and science. Additionally, students often underestimate the extent of statistics in these subjects (Ruggeri et al., 2008). As a consequence, statistics anxiety is supposed to lead to manifold problems over the course of students' statistics education. Students who experience higher levels of statistics anxiety are assumed to be more likely to procrastinate learning, e.g., to postpone writing term papers, to study for examinations, or to keep up with the weekly readings (Onwuegbuzie, 2004). Also, statistics anxiety is assumed to be related to less time spent on learning and to less efficient learning and study strategies (Macher et al., 2011, 2013). In the examination itself, statistics anxiety is related to worry and rumination and consumes processing capacity that would be needed for task performance (Papousek et al., 2012; Macher et al., 2013).

Consequently, statistics anxiety often is regarded to be one of the most powerful negative factors of influence on performance in statistics courses (Onwuegbuzie and Wilson, 2003). However, is this really so clear-cut? The studies in which statistics anxiety as well as performance in the examination was measured show ambiguous results concerning the relationship between statistics anxiety and performance; correlations were at best moderate, more often weak and even zero-correlations were found. Therefore, one may critically ask whether statistics anxiety really influences performance in statistics courses, and what implications this has for attaining statistical literacy. The present article takes up this question, scoping evidence from studies where statistics anxiety and performance were measured, then looking at the contribution of various indicators of performance, concluding with arguments on relevant mechanisms and implications.

## Relationship between statistics anxiety and performance

Evidence relating statistics anxiety with performance should be analyzed carefully because often the concept of statistics anxiety is defined and measured very imprecisely. Various studies and measurement instruments subsume variables such as academic self-concept or attitudes toward statistics under the term “statistics anxiety” (Elmore et al., 1993; Zanakis and Valenzi, 1997; Onwuegbuzie, 2004; Hanna et al., 2008). These variables, however, are not to be equated with statistics anxiety as an emotion characterized by feelings of tension, worried thoughts, mental disorganization, physiological arousal etc. in statistical contexts such as taking a test or dealing with statistical content (Papousek et al., 2012).

This scoping identified 11 studies that investigated the correlation between examination performance and statistics anxiety using an appropriate definition for anxiety. Five studies found non-significant correlations with values ranging from 0 to *r* = −0.20: Birenbaum and Eylath (1994) with first- and second-year students in education; Chiesi and Primi (2010) with measures of statistics anxiety and mid-course and final grades in a sample of psychology students; Lacasse and Chiocchio (2005) with students in a psychometrics class; Macher et al. (2013) with second-term psychology students; and Nasser (2004) with students in education. Macher et al. (2011) found a significant correlation of *r* = −0.21 in a sample of first-term students; Fitzgerald et al. (1996) report similar values. An exception to these results is the study by Lalonde and Gardner (1993), who found bivariate correlations of up to *r* = −0.49, but such findings have not been replicated since.

Three studies argued that the relationship between performance and statistics anxiety depended on students' academic background or the instructional context. Bell (2003) found zero-correlations for business students who started their university education immediately after leaving school and moderate, yet significant correlations for students who started their university education at an older age and after some vocational experiences. Bell (2001) also found significant correlations (up to *r* = −0.35) in courses which lasted a whole term in contrast to shorter courses (e.g., summer schools). Keeley et al. (2008) found significant relationships when the exam was more complex and challenging, thus difficulty is a potential moderator of the relationship. However, the highest correlation between anxiety and performance did not exceed *r* = −0.40.

## Statistics anxiety in comparison to other predictors of performance

Altogether, these low to modest correlations cast some doubt on the influence of statistics anxiety on performance. Thus, the question arises: are there better predictors for performance than statistics anxiety?

Few of the 11 studies described above have measured statistics anxiety and performance together with other predictors for performance. This is made complicated by the lack of uniform testing for acquiring statistical skills and arguments that new thinking is necessary in statistical assessment more generally (Ruggeri et al., 2011).

Some studies focused on cognitive variables. For example, inductive reasoning and high school mathematics grade were significantly related to statistics achievement in Birenbaum and Eylath (1994), basic mathematical abilities in Chiesi and Primi (2010). Mathematical ability and the level and number of courses in mathematics in school were related to performance in Lalonde and Gardner (1993) (but less than statistics anxiety). Other studies investigated the academic self-concept and/or interest in statistics. Academic self-concept is related to the actual competence in a field (Marsh and Yeung, 1997) and to more efficient learning strategies. Similarly, greater interest is linked to more time spent on tasks in a domain as well as to higher performance. In studies by Keeley et al. (2008), Macher et al. (2011, 2013), and Nasser (2004), academic self-concept and performance were significantly related. Macher et al. (2011, 2013) also connected these to interest and performance. In these two studies, self-concept and interest correlated with values between *r* = 0.21 and *r* = 0.34 with performance in the examination and showed stronger correlations with performance than statistics anxiety (Macher et al., 2011, 2013).

Generally, prior knowledge and variables that are related to prior knowledge but also self-concept and interest are strong predictors for future achievement (Marsh and Yeung, 1997). Studies which investigated the combined relationship between these variables, statistics anxiety, and performance show negative relations to anxiety but positive ones to performance (Lalonde and Gardner, 1993; Chiesi and Primi, 2010; Macher et al., 2011, 2013). These studies suggest to look not only at correlations but to investigate the interrelations between several variables.

## Mechanisms linking statistics anxiety to performance

The cognitive-interference approach (Eysenck et al., 1987) proposes a direct link between anxiety and performance in an examination: Anxiety leads to increased attentiveness to task-irrelevant aspects and thus subtracts cognitive resources from the examination task at hand. The deficit approach proposes an indirect link (Musch and Bröder, 1999): lower academic achievements are attributed to adverse learning behaviors prior to an examination. Students with high statistics anxiety may invest less effort and time for learning, use less efficient learning strategies, and consequently may be ill-prepared for examinations. Yet, both approaches cannot explain zero-correlations between statistics anxiety and performance.

**Figure 1:**
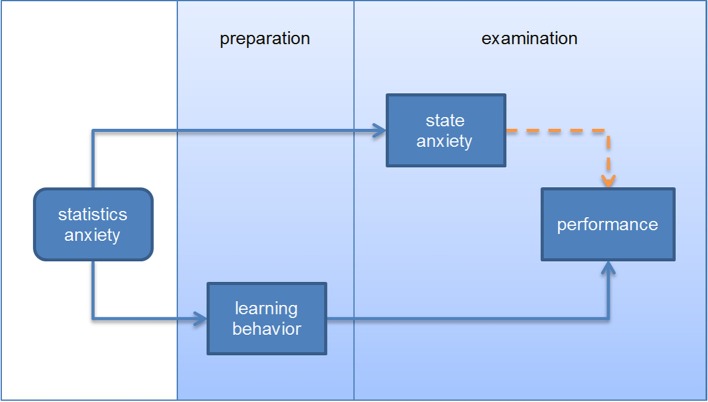
**The two-fold effect of statistics anxiety on performance**.

Findings of Macher et al. (2013) may explain these zero-correlations: Prior to the examination, statistics anxiety as well as other predictors for performance were measured (self-concept in mathematics, interest in statistics, etc.). Additionally, students rated their state anxiety twice during the examination: immediately before and in the middle of the examination. As expected, self-concept in mathematics and interest were related negatively to statistics anxiety and positively to performance. Results of the structural equation model pointed to a suppression effect between statistics anxiety and performance. The bivariate correlation between statistics anxiety and performance was around zero. Then two indirect effects with opposite signs for the correlations between statistics anxiety and two mediator variables could be observed: via state anxiety experienced immediately before and during the examination, statistics anxiety had a small but significant negative influence on performance. Statistics anxiety seemed to initiate a high level of state anxiety at the beginning of the examination which then was (at least to a larger degree) responsible for the maintenance of a high anxiety level throughout the examination. But statistics anxiety also had a small but significant positive influence on performance (probably through more efficient learning behaviors and/or more time spent on learning) (see Figure 1). Macher et al. (2013) also considered the possibility of a curvilinear relationship between statistics anxiety and performance but found no empirical evidence for this assumption. Students with extreme levels of statistics anxiety probably do not consider to study a subject with larger amounts of statistics or already fail the university entrance exams or drop out. Thus, a linear relationship should describe a student sample better.

The two-fold effect of anxiety may be explained by students' motivational goals in an educational setting: according to expectancy-value theory (Pekrun, 1988), test anxiety—and presumably statistics anxiety as well—usually reduces the motivation to approach an evaluative or a learning context because learners expect negative feelings and failure. In that instance, anxiety impairs performance by avoidance behavior such as reduced effort and less time spent for learning or avoiding the examination at all.

Depending on the situational context, such failure-avoidance motivation may have different impacts on effort motivation. In situations where effort avoidance lacks negative consequences (e.g., in laboratory settings), individuals may avoid failure by not exhibiting any achievement behavior and thus prevent the risk to fail. However, in many instructional contexts, students who do not invest sufficient effort and time for learning will face severe consequences such as failing the examination. The severity and unpleasantness of these consequences outweigh negative feelings in the preparation phase and the examination. In that case, anxiety strengthens positive extrinsic achievement-related effort motivation by the intention to avoid failure (Pekrun, 1988). Students with high degrees of statistics anxiety may experience debilitating levels of anxiety in the examination, but they should be motivated to invest effort in the examination preparations and to show appropriate learning behaviors for the examination. Thus, negative effects in the examination due to worry and rumination can be outweighed by enhanced effort in the preparation phase. In that sense, one could regard statistics anxiety as a “blessing in disguise.” Birenbaum and Eylath (1994, p. 96) also explain zero-correlations in their study with the assumption “students with high levels of statistics anxiety may have worked harder and suffered more in order to earn the same grade as their less anxious counterparts.”

Expectancy-value theory also explains results such as significant correlations between statistics anxiety and performance in difficult and zero-correlations in easier examinations (Keeley et al., 2008): When students judge their chances for success positively (in not too difficult examinations) they are more willing to invest effort and time. Similarly, students with a positive self-concept in statistics rate their chances to succeed positively (and most probably also have a higher prerequisite knowledge) and are more likely to exhibit effective learning behaviors.

## Conclusions

Taken together, these results suggest that the influence of statistics anxiety may differ over the course of learning, with prior positive influences and negative influences of state anxiety in the examination. Future research should take such two-fold effects into account and investigate the influence of statistics anxiety within a framework of variables and within a longitudinal design, creating the possibility for effective teaching interventions. Depending on factors such as the self-assessment of their abilities, the importance of a course, expectancies of failure and its consequences, anxiety may reduce or enhance motivation. Furthermore, there is a lack of studies that investigated the immediate effects of anxiety in an examination together with antecedents and consequences of anxiety. Research designs are recommended that take these variables and their long-term interactions into account.

## Author contributions

All authors of this article fulfilled the four requirements listed in “Frontiers of Psychology” (http://journal.frontiersin.org/journal/psychology#author-guidelines):

- Substantial contribution to the conception or design of the work; or the acquisition, analysis, or interpretation of data for the work; AND
- Drafting the work or revising it critically for important intellectual content; AND
- Final approval of the version to be published; AND
- Agreement to be accountable for all aspects of the work in ensuring that questions related to the accuracy or integrity of any part of the work are appropriately investigated and resolved.

## Conflict of interest statement

The authors declare that the research was conducted in the absence of any commercial or financial relationships that could be construed as a potential conflict of interest.
